# Attenuation of skin injury by a MARCO targeting PLGA nanoparticle

**DOI:** 10.1038/s41536-024-00381-z

**Published:** 2024-12-06

**Authors:** Ummiye V. Onay, Dan Xu, Dauren Biyashev, Spencer T. Evans, Michael M. Demczuk, Tobias Neef, Joseph R. Podojil, Sara Beddow, Nathan C. Gianneschi, I. Caroline Le Poole, Stephen D. Miller, Kurt Q. Lu

**Affiliations:** 1 Department of Dermatology, Feinberg School of Medicine, Northwestern University, Chicago, IL USA; 2 Department od Microbiology-Immunology, Feinberg School of Medicine, Northwestern University, Chicago, IL USA; 3 Cour Pharmaceutical Development Company, Northbrook, IL USA; 4 Department of Chemistry, Northwestern University, Evanston, IL USA; 5 International Institute of Nanotechnology, Simpson-Querrey Institute, Chemistry of Life Processes Institute, Lurie Cancer Center, Northwestern University, Evanston, IL USA; 6 Department of Materials Science and Engineering, Northwestern University, Evanston, IL USA; 7 Department of Biomedical Engineering, Northwestern University, Evanston, IL USA

**Keywords:** Regenerative medicine, Translational immunology, Drug development

## Abstract

Cutaneous exposure to the DNA alkylating class of chemotherapeutic agents including nitrogen mustard (NM) leads to both skin injury and systemic inflammation. Circulating myeloid subsets recruited to the skin act to further exacerbate local tissue damage while interfering with the wound healing process. We demonstrate herein that intravenous delivery of poly(lactic-*co*-glycolic acid) immune-modifying nanoparticles (PLGA-IMPs) shortly after NM exposure restricts accumulation of macrophages and inflammatory monocytes at the injury site, resulting in attenuated skin pathology. Furthermore, PLGA-IMPs induce an early influx and local enrichment of Foxp3^+^ regulatory T cells (Treg) in the skin lesions critical for the suppression of myeloid cell-pro-inflammatory responses via induction of IL-10 and TGF-β in the cutaneous milieu. Functional depletion of CD4^+^ Tregs ablates the efficacy of PLGA-IMPs accompanied by a loss of local accumulation of anti-inflammatory cytokines essential for wound healing. Thus, in severe skin trauma, PLGA-IMPs may have therapeutic potential via modulation of inflammatory myeloid cells and regulatory T lymphocytes.

## Introduction

Being the largest organ in the body, the skin is commonly exposed to numerous environmental insults and works with immune processes to resolve acute injury^1^. Circulating innate immune cells respond to injury-induced chemokines and danger signals by infiltrating damaged tissue to clear debris and initiate tissue repair^1^. However, chemokines and inflammatory cytokines produced by activated inflammatory cells, often lead to bystander tissue damage resulting in amplified immune-mediated pathology^2^.

Nitrogen mustard (NM) was the first chemotherapeutic agent discovered in 1942 for the treatment of Hodgkin’s lymphoma and other hematologic cancers^3,4^. Along with its numerous derivatives, this class of alkylating agents bind to and crosslink DNA and are frequently used in cancer therapy^5^. However, drug-induced toxicities are common and often necessitate treatment cessation. NM treatment for skin lymphoma (mycosis fungoides) can result in severe dermatitis and blister formation^6^. Based on historical accounts of victims exposed to the infamous, related sulfur-containing compound, mustard gas, these cytotoxic effects can cause severe injury and death. We and others have shown that the damage resulting from exposure to alkylating agents is not solely attributable to the “1st hit” direct cytotoxicity of the chemical agent^7–9^. Damaged keratinocytes in the epidermal barrier release high levels of TNF-α, iNOS, ROS, chemokines and proteolytic enzymes creating a highly pro-inflammatory milieu^7,10–14^. Subsequent recruitment of innate immune cells, specifically inflammatory monocytes and macrophages, to the inflamed site represents an important “2^nd^ hit” that further exacerbates tissue damage^7,10–14^.

In prior studies, we demonstrated that clodronate-mediated macrophage depletion prevented macrophage migration to the bone marrow and subsequent iNOS-mediated innate immune cell recruitment, thereby suppressing skin inflammation and necrosis after NM application^7^. These experiments provided the premise for the current studies, wherein we sought to determine whether the above findings could be achieved with a translationally relevant therapeutic intervention. To test this hypothesis, we employed a systemic administration of carboxylated biodegradable immune-modifying nanoparticles (<1 μm in diameter), composed of poly(lactic-*co*-glycolic acid) (PLGA-IMPs). Carboxylation promotes particle uptake. In the setting of systemic sclerosis (SSc), we reported that biodegradable PLGA particles can alter the activation pattern and trafficking of inflammatory monocytes, wherein PLGA-IMPs are engulfed by circulating inflammatory monocytes via the macrophage receptor of collagenous structure (MARCO) scavenger receptor^15^. Phagocytic myeloid cells, including Inflammatory monocytes, are sequestered in the spleen where they undergo apoptosis and are thus diverted away from the site of acute injury. While the treatment initially depletes myeloid cells and may appear to be immunosuppressive, the functionality and mechanism of action of PLGA nanoparticle treatment is more complex. In both CNS viral infection and lung infection mouse models, treatment with PLGA nanoparticles inhibits inflammatory monocyte infiltration into the site of infection, thereby decreasing inflammatory immune cell-induced tissue damage, while also allowing for a more controlled virus-specific immune response such that viral clearance and immunological memory are engendered^15,16^. Further support for the ability of PLGA nanoparticle treatment to inhibit inflammatory monocyte-induced tissue damage has been shown in models of ischemic reperfusion injury^15^, spinal cord injury^17^, and traumatic brain injury^18^. In the current studies, we found that that PLGA-IMP treatment mitigated NM-induced skin injury by inhibiting accumulation of inflammatory monocytes via a mechanism involving enrichment of Tregs in the wound bed.

## Results

### Therapeutic PLGA nanoparticle treatment attenuates NM-induced eschar formation and dermal pathology

**Fig. 1:**
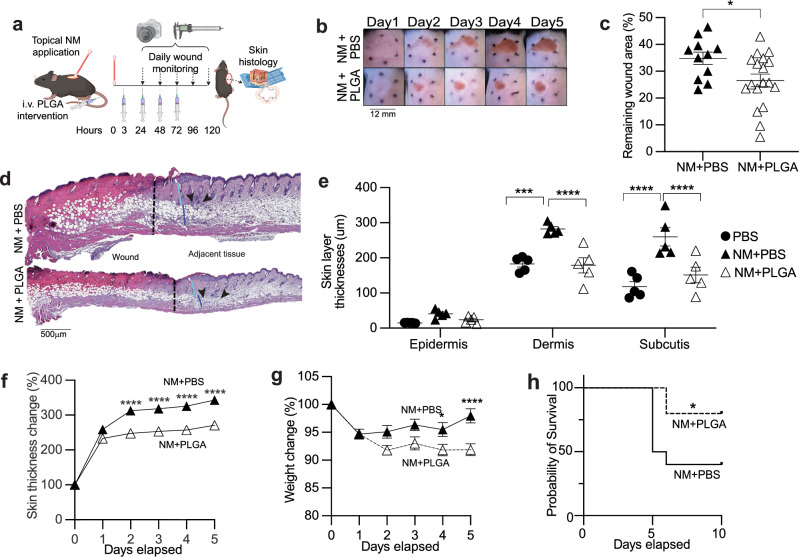
PLGA-IMP treatment significantly ameliorates inflammation in mice with NM-induced skin wounds. The experimental setup (**a**) cartoon is prepared with BioRender. Daily treatment with PLGA-IMPs significantly reduces (**b**) eschar formation, (**c**) wound area, (**f**) skin thickness and (**g**) weight change by day 5 post NM insult. **d** shows representative hematoxylin and eosin-stained histological images of the punch biopsies taken on Day 3 post NM insult. Vertical black line indicates the wound border, white, light blue and dark blue lines indicate the thickness of epidermis, dermis and subcutis layers, respectively. Black arrow heads point at the swarm of infiltrating immune cells. **e** shows epidermal, dermal and subcutis layer thickness measurements using Image J. **h** is the Kaplan-Meier graph showing the PLGA-IMPs’ effect in protecting the mice from systemic effects of high dose (4%) NM (*n* = 10 per group). *p < 0.05, **p < 0.01, ***p < 0.001, ****p < 0.0001. BioRenderTM was used to create cartoon visualizations of experimental setups in (**a**).

Skin exposure to toxic doses of NM results in not just skin pathology but also suppression of blood and bone marrow cell populations, hence its use for treating leukemia and lymphoma. In a lethal exposure murine model, we have observed NM-induced acute pancytopenia, and that activated macrophages play a major role in local skin and bone marrow destruction^7,19^. Furthermore, in a randomized double-blinded placebo-controlled clinical trial of healthy humans patch-tested in vivo with a trace amount of FDA-approved topical NM gel, we observed upregulation of numerous macrophage chemotactic cytokines^20^ in skin biopsies. To determine the pathogenic contribution of myeloid cells and lymphocytes to NM-induced skin burn, we leveraged the anti-inflammatory properties of PLGA immune-modifying nanoparticles (PLGA-IMPs). We have reported that PLGA-IMPs are engulfed by inflammatory myeloid cells by the MAcrophage Receptor with COllagenous structure (MARCO) and regulate inflammatory myeloid cell trafficking and function, thereby mitigating tissue damage and promoting healing in a wide variety of acute inflammatory conditions^15,16^. Intravenous PLGA nanoparticle delivery (1 mg/mouse) was initiated 2 h post NM exposure at a dose of 11 mg/kg (Fig. 1a). Daily administration of PLGA nanoparticles for up to 4 days was well-tolerated with no measurable signs of toxicity.

As expected, in the sub-lethal NM exposure model, there were prominent local tissue architectural alterations and immune activation in the skin, including erythema associated with hemorrhagic crust that remained enlarged resulting in eschar formation, weight loss, erosion of the epidermal layer, disruption of hair follicles, and tissue damage (Fig. 1b–g). Hallmarks of NM-induced tissue damage were all markedly attenuated by PLGA treatment. Eschar formation was significantly reduced as measured by both wound size and skin thickness (Fig. 1b, c, f). Strikingly, treated mice displayed a milder histological phenotype with diminished skin necrosis that was limited to the epidermis while preserving the subcutaneous tissues and hair follicles (Fig. 1d, e). In the more toxic LD_60_ exposure model, daily PLGA treatment rescued 50% of mice from succumbing to the systemic injury (Fig. 1h).

### PLGA nanoparticle treatment restricts early accumulation of activated myeloid cells in the skin lesion

**Fig. 2:**
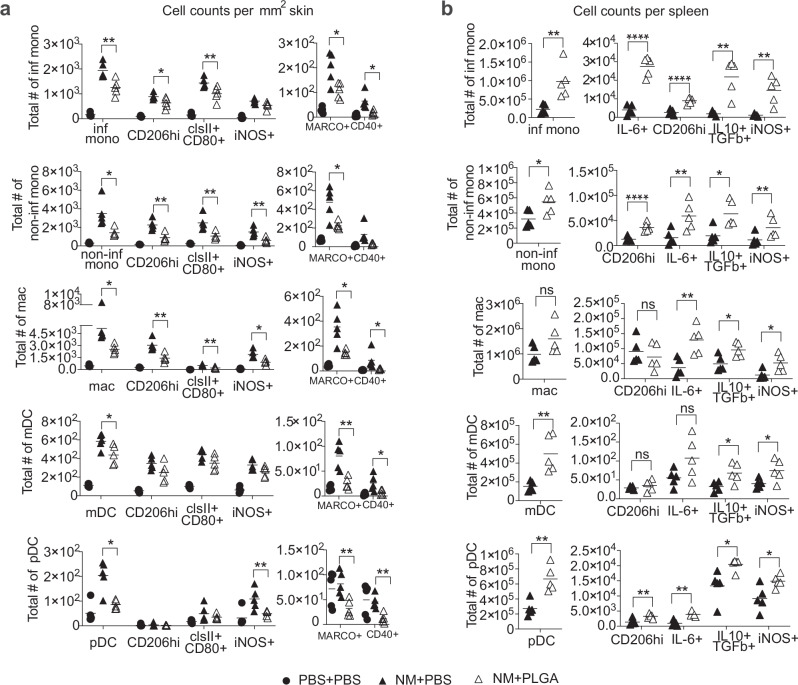
PLGA-IMP treatment significantly reduces the infiltration/accumulation of inflammatory cells in NM-induced cutaneous wounds. **a** show the reduction in total number of APCs and myeloid cells per mm^2^ of skin, as well as the change in activation markers expressed by these cells following PLGA-IMP treatment. **b** shows the changes observed in spleen. *p < 0.05, **p < 0.01, ***p < 0.001, ****p < 0.0001.

Modulation of acute immune infiltration and activation shortly after NM exposure orchestrates long-term outcome^7^. Our time course study showed stable numbers of CD45^+^ immune cells between day 2 (D2) and day 5 (D5) post NM exposure (Supplementary Fig. 1). We thus focused on day 3 (D3), a time point consistent with our published clinical trial data collection (NCT02968446^20^), to ascertain the effects of PLGA nanoparticles on the immune infiltrates by flow cytometric analysis (Fig. 2, Supplementary Fig. 2). Three doses of PLGA nanoparticles at 3, 24 and 48 h post NM application significantly reduced eschar-infiltrating myeloid cells while redirecting the activated leukocytes to the spleen (Fig. 2a, b). Antigen-presenting cell subsets that were averted from the eschar comprised inflammatory monocytes, non-inflammatory monocytes, macrophages, mDCs, and pDCs (Fig. 2a). Notably, the absolute numbers of each of these myeloid subsets expressing activation markers (MHC II, CD80, iNOS, CD206, and CD40) were also significantly attenuated by PLGA-IMP treatment (Fig. 2a). Interestingly, separate macrophage subsets characterized by expression of iNOS and CD206 respectively, were similarly reduced. It is noteworthy that there was a significant loss of MARCO^+^ myeloid cells consistent with histological analysis and corroborating the clinical data (Fig. 2a). In contrast to the attenuation of dermal infiltrates, significant accumulation of the corresponding myeloid cells in the spleen was observed (Fig. 2b). PLGA nanoparticle-induced trafficking redirection of myeloid cells and sequestration of activated APC in the secondary lymphoid organs is apoptosis dependent (manuscript in preparation). Consequently, reduced immune pathology was observed in the peripherally inflamed tissue, including the skin due to the splenic retention of apoptotic APCs. Remarkably, myeloid cells dually producing IL10^+^/TGF-β^+^ were enriched in the spleen.

### Enrichment of regulatory CD4^+^ and CD8^+^ T lymphocytes in NM-induced skin lesions following PLGA nanoparticle treatment

**Fig. 3:**
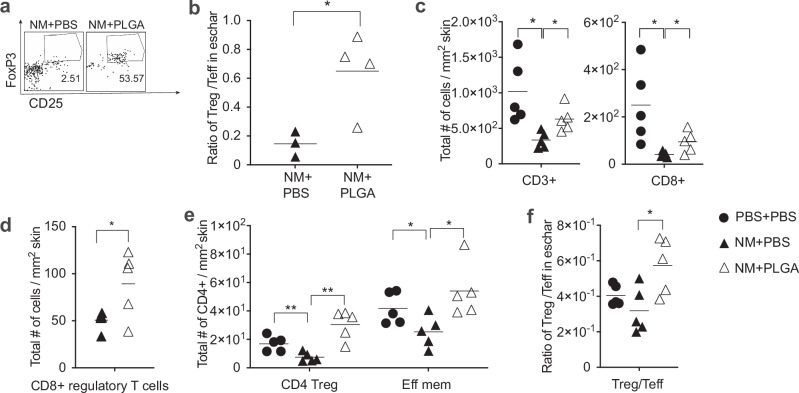
PLGA-IMP treatment Induces early accumulation of Tregs in NM-induced cutaneous wounds. PLGA-IMP treatment induces early accumulation of Tregs in NM-induced cutaneous wounds. **a** shows the increased frequency Tregs at the wound site following 2 doses of PLGA-IMP intervention. **b**, **f** show the significant increase in the Treg/Teff ratio after 2 and 3 doses of PLGA-IMP intervention, respectively. **c**, **d**, **e** show significant variation of T cell population and their functional markers in the wound, following 3 doses of PLGA-IMP intervention. *p < 0.05, **p < 0.01, ***p < 0.001, ****p < 0.0001.

In addition to the myeloid compartment, we also enumerated lymphocyte subsets, most notably Tregs, Teff CD4^+^, CD8 T cells, B cells, and natural killer cells in NM-induced skin lesions following PLGA IMP treatment (Fig. 3, Supplementary Fig. 3). As early as 48 h post NM exposure, with only 2 doses of PLGA nanoparticles delivered, the frequency of dermal Tregs in the treated mice increased 20-fold over control levels (Fig. 3a). More importantly, the Treg/Teff ratio was also significantly augmented suggesting a potential protective role of Tregs in the inflammatory milieu early after PLGA IMP treatment (Fig. 3b). We therefore sampled a few time points to ascertain the enrichment of this major regulatory T cell population in the eschar. Compared to D2, the frequency of Treg was maintained on day 3 (D3) (Fig. 3e). Despite the observation that CD4^+^ Teff cells were also increased in the skin, the ratio of Treg/Teff remained significantly higher in treated mice (Fig. 3f). In addition to the CD4^+^ Tregs, we also observed a significant increase in a less defined and poorly understood population of heterogenous regulatory CD8^+^ T cells defined by their surface expression of CD8^+^CD122^hi^CD49d^+^ (Fig. 3c, d). CD8^+^ Tregs can exert regulatory functions either via direct cytotoxicity by lysing CD4^+^ Teff and T follicular helper (Tfh) T cells via perforin lysis or via Fas-FasL-mediated suppression of activation and proliferation of target cells. Together, CD4^+^ and CD8^+^ Treg subsets contributed to the increase in total number of CD3^+^ lymphocytes in the eschar (Fig. 3c).

**Fig. 4:**
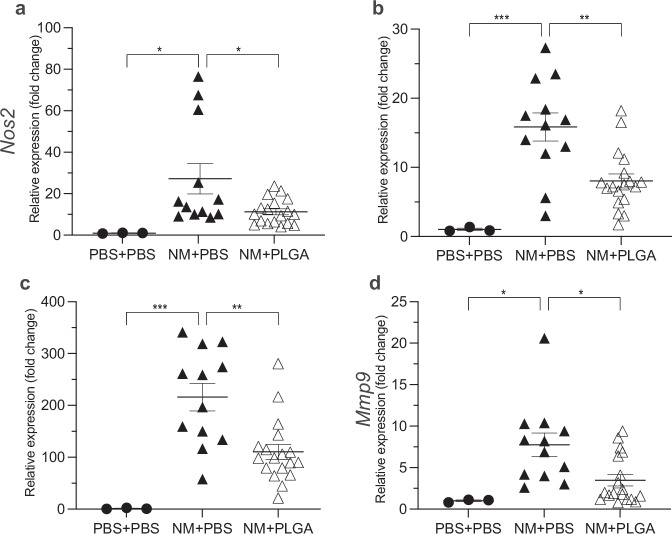
PLGA-IMPs significantly attenuate the inflammatory gene expression profile in NM-induced skin wounds. PLGA-IMPs significantly attenuate inflammatory gene expression profile in NM-induced skin wounds. Real time quantitative PCR of day 5 skin shows significant increase in the expression of (**a**) Nos2, (**b**) Tnfa, (**c**) Il-1b, and (**d**) Mmp9 in NM treated cohort compared to the non-treated cohort, as well significant attenuation of inflammatory gene expression following PLGA-IMP treatment. *p < 0.05, **p < 0.01, ***p < 0.001, ****p < 0.0001.

The attenuation of proinflammatory myeloid cells along with a boost in regulatory T lymphocytes created an anti-inflammatory milieu within hours of PLGA nanoparticle delivery. Consistent with the intracellular cytokine production shown in Fig. 2a, qPCR evaluation of skin lesion tissue homogenates revealed approximately a 50% reduction in expression of pro-inflammatory cytokine genes, including *Nos2* (Fig. 4a)*, Tnfa* (Fig. 4b), and Il-1b (Fig. 4c), as well as *Mmp9* (Fig. 4d). Changes in these cytokines and MMP would consequently modulate the trafficking and function of activated myeloid cells.

### Functional inactivation of CD4^+^ CD25^+^ Tregs abrogates the efficacy of PLGA nanoparticles

**Fig. 5:**
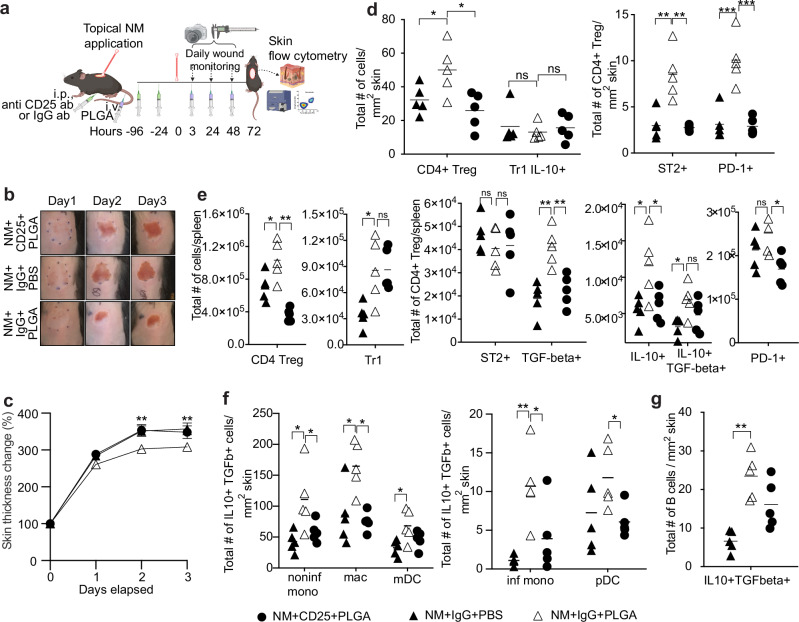
CD25^+^ Treg depletion reverses the wound healing effect of PLGA-IMP nanoparticles. The experimental setup (**a**) cartoon is prepared with BioRender. **b**, **c** show the wound monitoring and skin thickness measurements, respectively. **d**, **e** show significant reversal of T cell populations and their increased expression of activation markers in the wound and spleen, respectively, following anti-CD25 intervention. **f**, **g** show the effect of anti-CD25 intervention on the myeloid cell populations. *p < 0.05, **p < 0.01, ***p < 0.001, ****p < 0.0001. BioRenderTM was used to create cartoon visualizations of experimental setups in (**a**).

To delineate a functional requirement for CD4^+^CD25^+^FoxP3^+^ Tregs in the control of NM-induced eschar formation and pro-inflammatory immune activation, we inactivated Treg function by injecting anti-CD25 monoclonal antibody (Clone PC61) prior to NM exposure and PLGA-IMP treatment (Fig. 5a)^21^. By D2, the PLGA/control IgG-treated group already exhibit attenuation of eschar formation, whereas eschar formation in the PLGA/anti-CD25-treated group, in the absence of CD4^+^ Tregs, was indistinguishable from the NM-PBS control group (Fig. 5b). In addition, attenuation of dermal thickening was reversed in the absence of CD4^+^ Treg function (Fig. 5c). Anti-CD25 antibody only affects the CD4^+^ Foxp3^+^ Treg compartment leaving other CD4^+^ regulatory T cell subsets, such as the Type 1 regulatory (Tr1) cells, intact in the skin and the spleen (Fig. 5d, e). The loss of Treg function is measurable by the expression of surface markers, ST2 (functional receptor for IL-33), Programmed Death-1 (PD-1), IL-10, and Transforming Growth Factor β (TGF-β) critical for the induction and maintenance of suppression (Fig. 5d, e). Remarkably, the absence of functional CD4^+^ Tregs was paralleled by loss of a wide range of myeloid cells, including inflammatory and non-inflammatory monocytes, macrophages, pDCs, and mDCs, in the skin lesion (Fig. 5f). Interestingly, anti-CD25 administration failed to ameliorate the numbers of IL-10 + TGF-β + B cells (Fig. 5g). These immune regulatory APCs were dual producers of IL-10 and TGF-β, which are implicated in tolerance induction, as well as in downregulation of MHC class II and costimulatory molecules via STAT/JAK pathways.

### Dermal infiltration of MARCO^+^ myeloid phagocytes in NM-exposed skin of healthy humans and mice

**Fig. 6:**
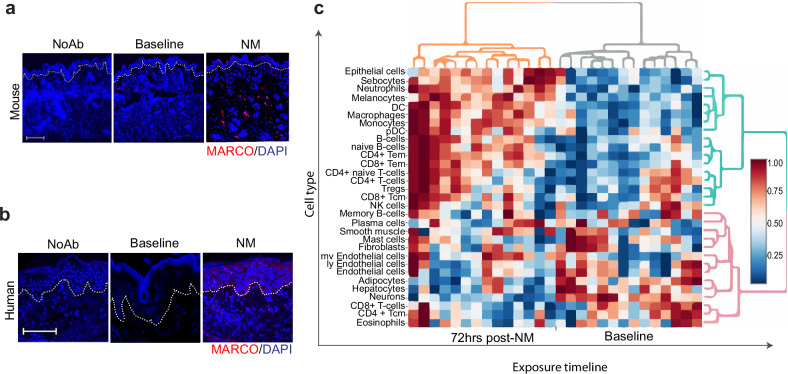
Exposure of skin to nitrogen mustard results in the influx of MARCO-expressing cells. Immunofluorescence staining of MARCO (red) in mouse (**a**) and human (**b**) skin sections. Mice were treated with nitrogen mustard and human subjects with Valchlor. Nuclei were stained with DAPI (blue). Dotted line indicates epidermal—dermal junction. Images were taken at 20× magnification, scale bar is equal to 100 µm. **c** shows the cell enrichment analysis of human skin biopsies taken at baseline and 72 h post-NM (Valchlor) treatment.

Our previous and current (Fig. 2) flow cytometry analyses indicate a major pathologic contribution of activated monocytes and macrophages expressing the MARCO receptor to acute inflammation in a variety of acute inflammatory conditions^16^. In addition, we have shown that PLGA-IMP treatment significantly decreases the accumulation of MARCO^+^ inflammatory myeloid cells in inflamed tissues. Most relevant to NM-induced skin injury, we recently reported the identification of a novel MARCO^+^ fibrosis-driving macrophages and inflammatory monocytes in skin lesions of a mouse model scleroderma induced by the chemotherapeutic drug, bleomycin (BLM)^16^. NM and BLM share common features of dermal immune activation, as well as induction of fibrosis following cutaneous exposure. To confirm and translate our flow cytometry results seen in Fig. 2, we next examined skin exposed to NM for accumulation of MARCO-expressing mononuclear cells. Here, we show that in both mice and human subjects, exposure in vivo to topical NM (0.5% vs. 0.016%, respectively) results in accumulation of MARCO^+^ infiltrates, with the latter being a significantly less concentrated amount of NM (Fig. 6a, b). De-identified human skin biopsies were obtained from our published clinical trial (NCT02968446^20^) in which healthy subjects were patch-tested to a trace amount of topical NM in the form of FDA-approved mechlorethamine 0.016% gel brand Valchlor®.

Given these findings, we further interrogated the transcriptomes of 14 sets of paired human skin biopsies at baseline (pre-exposure) and 72 h post-NM (Valchlor) exposure to ascertain immune infiltrates and further delineate cellular subsets in human dermal lesions. Baseline skin biopsies serve as controls and are taken from the same individuals at a distant anatomic site. The RNAseq data revealed an array of highly enriched myeloid phagocytes in NM-exposed dermis consistent with the murine models (Figs. 2, 3). In human skin a myriad of myeloid cells including neutrophils, conventional dendritic cells (cDC), plasmacytoid DC (pDC), and monocytes were detected (Fig. 6c)^20^. Contrary to the previously held notion of myeloid-driven skin damage after NM exposure, we also detected a dynamic repository of antigen-experienced memory T lymphocytes, particularly effector memory (Tem) CD4^+^ helper and CD8^+^ cytotoxic T cells. In contrast to the Tem subsets, the central memory (Tcm) and naïve T cell compartment remain largely unperturbed post-NM exposure. Interestingly, T cells were not the only lymphocytic cell type detected. We observed B cell enrichment in the skin biopsies which has not been previously reported. In contrast to the T cells, the level of terminally differentiated Ag-experienced plasma cells was comparable to baseline. However, total B cells, particularly naïve B cell subsets that retain differentiation capacity were elevated following NM exposure (Fig. 6c). Collectively, these results suggest very similar mechanisms are operative in the skin of mice and humans exposed to NM.

### Immunomodulatory effect of PLGA-IMP was ablated in MARCO-deficient (MARCO^−/−^) mice

**Fig. 7:**
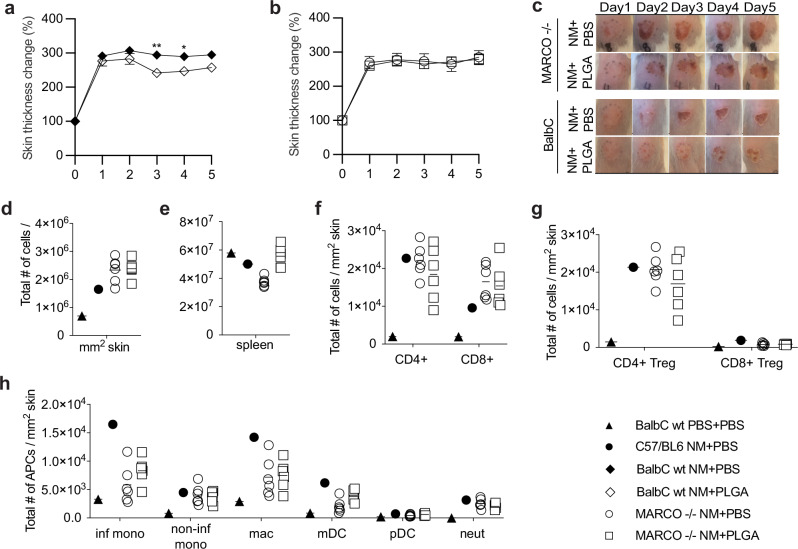
The efficiency of PLGA-IMP intervention is ablated in MARCO knockout mice. PLGA-IMP treatment significantly reduces skin thickness in wt Balb/c mice (**a**) but not in the MARCO −/− mice (**b**). Eschar formation is not reduced with daily PLGA treatment in MARCO −/− mice (**c**). **d**–**h** show flow cytometry analysis of immune infiltrating cells.

To further ascertain the contribution of MARCO+ inflammatory monocytes and macrophages in the PLGA-IMP-mediated amelioration of NM-induced skin burn, we adopted a MARCO knockout (MARCO^−/−^) mouse model. Because we only employed the MARCO^−/−^ mice on the BALB/c background, we first conducted a comparison study between wt BALB/c and wt C57BL/6 mice which showed that NM-induced wound severity, timeline, and immune activation were comparable between the two strains (Figs. 7a, c, 1f). We then exposed MARCO^−/−^ mice to NM and treated them with PLGA-IMP or PBS as the control. Remarkably, the immunomodulatory effect of PLGA-IMP was ablated in the MARCO^−/−^ strain (Fig. 7b, c, Supplementary Fig. 4). Analysis of the immune infiltrates in the NM-injured skin revealed that in the absence of MARCO, PLGA-IMP failed to reduce total immune infiltrates in the eschar (Fig. 7d). The retention of immune cells in the spleen observed in wt B6 and BALB/c mice was not detected in the MARCO^−/−^ strain (Fig. 7e). Regulatory T cells were absent in the eschar (Fig. 7f, g) consistent with the inability IMP treatment of MARCO knockout mice to reduce inflammatory infiltrates in the lesional skin (Fig. 7h). These findings thus further emphasize the importance for targeting of MARCO^+^ myeloid cells in the clinical efficacy of PLGA-IMP-induced immune regulation.

## Discussion

In this study, we identified previously undefined myeloid cells that contribute to the pathology of NM-induced acute inflammatory injury. Our findings implicate poorly understood myeloid cell subsets characterized by the surface expression of the scavenger receptor MARCO, as important mediators of acute dermal injury. We provide the first evidence that MARCO^+^ monocytes, macrophages, and DCs accumulate in dermal lesions in human subjects and mice exposed to a sublethal dose of NM. Although MARCO was identified several decades ago, its function, pathological contribution to, and therapeutic potential in NM burns has never been reported or examined. Targeted reduction of cutaneous infiltration of MARCO^+^ myeloid subsets by leveraging the immunomodulatory properties of carboxylated PLGA nanoparticles effectively attenuated skin pathology and immune infiltration to allow accelerated would healing after topical application of NM. There were much fewer myeloid cells in the skin at steady state compared to post NM-injury as shown in Fig. 2a. The majority of the tissue-resident myeloid cells do not actively proliferate but are replenished by blood-borne precursors. Therefore, the majority of myeloid cells detected in the eschar post NM-injury were blood-borne. The reduction of immune infiltrates in the eschar was not due to compromised vascularization as we found that the frequency of CD31 positive cells as determined by flow cytometric analysis was comparable in PBS control and NM-treated skin. In addition to modulation of MARCO^+^ APCs, a short course of systemically administered biodegradable PLGA-IMPs elicited robust accumulation of CD4^+^ and CD8^+^ regulatory T cells in the NM-induced injury site. Functional depletion of CD4^+^CD25^+^ Tregs abrogated the therapeutic efficacy of PLGA nanoparticles. The dose and efficacy of anti-mouse-CD25 antibody infusion to inactivate the Treg population without affecting CD4^+^Foxp3^-^ effector T cell activation/functions has been well established by our laboratory and others in a number of mouse models of autoimmune disease^21^. The overall outcome of anti-CD25 treatment was loss of immune suppression. Hence, we conclude that anti-CD25 treatment functionally inactivated Tregs in PLGA-IMP-treated mice exposed to NM, demonstrating the essential role of the regulatory T lymphocytes in mitigating NM-induced dermal injury.

We have previously reported the role of dermally activated macrophages in the exacerbation of bone marrow pathology after NM exposure^7^. The current study built upon this prior knowledge to demonstrate that the myeloid repertoire involved in the NM-induced injury is more comprehensive, comprising inflammatory and non-inflammatory monocytes, as well as various subsets of DCs. One of the major APCs are monocytes, a heterogeneous population in the circulation. Both humans and mice have at least two different monocyte subsets, inflammatory and non-inflammatory, differentiated by their distinct function and differential expression of surface markers. Inflammatory monocytes express Ly6C^hi^ (mouse) or CD14^+^CD16^-^ (human) and are specialized in transporting antigens to secondary lymphoid organs and accumulate at peripheral sites of inflammation where they can differentiate into macrophages or DCs^22^. In contrast, non-inflammatory monocytes reside mostly in peripheral tissues or the vasculature lumen and are involved in early responses to inflammation and tissue repair. In the skin, inflammatory monocytes are thought to continuously replenish dermal macrophages, and DCs^23^. In our model, dying cutaneous cells induced by NM exposure are taken up by MARCO-expressing macrophages. Subsequent cutaneous CCL22 expression can drive recruitment of Tregs to the exposure site whereas macrophages accumulating in the spleen drive the activation and accumulation of Tregs for distribution to the skin. In the presence of PLGA treatment-induced Tregs, monocytes can be driven toward an alternatively activated state with reduced production of pro-inflammatory cytokines, and downregulation of co-stimulatory molecules and MHC-class II.

In addition to monocytes and macrophages, we also detected DCs infiltrating sites of NM injury, which play roles in both initiating inflammatory responses while also releasing abundant levels of immunosuppressive molecules (e.g., IL-10, TGF-β, IDO, and vitamin D) to the spleen. Three major subsets of DCs, cDC1, cDC2, and pDC, have been identified in humans and animal models. pDCs are highly effective in type I IFN production in response to intracellular sensing of viral or self-nucleic acids via TLR-7 and -9, whereas cDC1 and cDC2 are specialized in processing and presenting exogenous antigens to naïve CD4^+^ and CD8^+^ T cells, respectively, leading to their activation^24^. Under tolerogenic conditions, these DC subsets may be programmed to induce FoxP3^+^ CD4^+^Treg, Tr1 Tregs, and CD8^+^ Tregs. Indeed, we observed that the number of pDCs and mDCs doubly producing IL-10^+^ and TGF-β^+^ of were significantly augmented in NM lesions after PLGA nanoparticle administration. There was a concomitant attrition of activated pro-inflammatory dermal mDCs and pDCs characterized by upregulation of MHC class II, co-stimulatory molecules (CD80/CD86), MARCO, CD40, and iNOS. Consequently, an enrichment of CD4^+^ and CD8^+^ Tregs in the skin and spleen was observed^24^. The critical role of DCs in the induction of tolerance following NM exposure merits further investigation to delineate the molecular pathways that can be potentially targeted for drug development.

In addition to the myeloid cells, we report for the first-time enrichment of cutaneous T and B lymphocytes in both humans and mice shortly after dermal exposure to NM. This raises the possibility of a contribution of B cells to tissue damage and subsequent injury propagation following dermal application of NM. Both RNAseq using human skin biopsies and flow cytometric analysis of mouse dermal punch biopsies revealed accumulation of effector memory (Tem), naïve, and regulatory T cells (Tregs), as well as B cells. PLGA nanoparticle intervention significantly augmented dermal Tregs that mitigate tissue damage by controlling myeloid cell-driven inflammation, modulating Tem function, and facilitating tissue repair^25^. We showed that Tregs isolated from the eschar predominantly expressed ST2, the IL-33 receptor, after PLGA treatment. IL-33, secreted mostly by nonhematopoietic cells and a member of IL-1 family cytokines, is considered an essential driver of Treg retention in peripheral tissues. Though T cell subsets were not individually identified, γδ T cells are known to be important in wound healing^26^. It is possible that FoxP3+ γδ2 T cells with suppressive function are among the Treg population with increased abundance in response to PLGA nanoparticles and associated TGFβ concentrations observed here^27^. Tregs that maintained effector functions in non-lymphoid tissues have been identified by the expression of ST2^28^. In addition to ST2, eschar infiltrating Treg also upregulated PD-1, another marker that plays a crucial role in the induction and maintenance of Tregs. Augmenting dermal Tregs can lead to amelioration of skin fibrosis in scleroderma patients^29^. Conversely, dysfunctional Tregs could contribute to exacerbation of various autoimmune diseases involving dermal pathology, such as scleroderma, psoriasis, and graft-versus-host diseases. This was partially due to reduced levels of IL-10 and TGF-β in the skin. An enhanced Treg/Teff ratio is often used as a benchmark for effective T cell-mediated immunoregulatory therapy. PLGA nanoparticle treatment preferentially skewed T cell response toward Treg induction. These findings suggest that PLGA nanoparticle treatment regulated both the Treg and Teff compartments, inhibiting NM-induced disease pathology, and facilitating the healing process.

We identified a previously unreported lymphocyte subset in the NM-exposed skin. Both the clinical trial and the mouse model revealed cutaneous enrichment of B cells in the skin lesion, particularly naïve B cells. This observation is quite unique because naïve B cells are generally found in secondary lymphoid organs, such as lymph nodes, in proximity to CD4^+^ helper T cells. Dermal resident B cells remain understudied, and their function is largely unknown. There is growing recent evidence associating dermal B cells, particularly the IL10^+^ and TGF-β^+^ subset, with skin homeostasis and repair by regulating would healing^30^. Significantly, PLGA nanoparticle treatment augmented IL10^+^TGF-β^+^ B cells in the skin lesions.

Other innate immune cells may also contribute to the immunosuppressive effects of PLGA nanpoparticles, including subsets of granulocytes and innate lymphocyte subsets. If specific subsets of innate immune cells are activated in response to PLGA particles, their effect might be subordinate to that of macrophages and Tregs since depletion of these sequentially involved subsets completely abrogates the therapeutic effect of the nanoparticles. However, it is possible that said immune cells translate signals provided by emigrating macrophages to induce Treg accumulation in the skin. Ongoing studies are currently addressing this possibility.

We do not anticipate that short-term PLGA-IMP treatment will interfere with cancer therapies as we have previously reported that treatment of mice with PLGA nanoparticles can modulate the anti-tumor immune response such that anti-tumor NK cell and CD8^+^ T cell responses are increased via the activation of the STING pathway^31,32^. The use of PLGA-IMP is under investigation for tumor microenvironment re-modeling and for its potential as an adjunct to classical immunotherapy for the treatment of tumors in a variety of mouse models.

In conclusion, the current study provides evidence that Tregs play a critical role in would healing and amelioration of NM-induced pathology via modulation of IL10^+^TGFβ^+^ myeloid cells - monocytes, macrophages, and DCs. Additionally, we demonstrate that MARCO^+^ APCs are potential targets for the treatment of NM-induced immunopathology. Significantly, we describe a biodegradable nanoparticle-based therapy with an outstanding safety profile that simultaneously modifies disease outcome^33^. This is the first study to report a novel therapeutic approach for treatment of NM exposure utilizing a nanomaterial strategy that targets multiple cell lineages contributing to eschar formation and progression.

## Methods

### Mice

Six- to eight-week-old C57BL/6J female mice (stock no: 000664) were purchased from Jackson Laboratories (Bar Harbor, ME). All animal studies have been approved by the Northwestern University IACUC.

### Wound induction

Mice in the control and experimental treatment groups were acclimated for 2 days before their dorsal area was shaved and chemically depilated using Nair hair removal gel while they were under inhalational anesthesia (VetEquip V1 small animal anesthesia system with isoflurane). Two days after depilation, mice were anesthetized via intraperitoneal injection (125–250 mg/kg) of freshly prepared and filter sterilized 1.25% solution of Avertin (Sigma T48402, pH 7). A 12 mm circular template was drawn on the depilated area and baseline skin thickness measurements were taken. A 0.5% solution of Mechlorethamine hydrochloride (nitrogen mustard, NM) (Sigma 122564) in 1.5% DMSO-PBS was applied into the template area in a chemical hood. Mice were kept under observation on a warming pad until the NM dried and were then returned to their cages. The cages were kept in a chemical fume hood for 2 h before they were returned to the mouse facility.

### PLGA particle preparation

Poly(lactic-co-glycolic acid) (PLGA) nanoparticles were obtained from COUR Pharmaceuticals (Skokie, IL) and prepared with proprietary formulations. Briefly, polymer was dissolved in a proprietary organic solvent, subsequently mixed with an aqueous surfactant before being sonicated to make a single oil-in-water emulsion. After removing the solvent by evaporation, PLGA nanoparticles of an average 500 nm in size and a zeta-potential of −75 mV were obtained. The resulting nanoparticles were washed and freeze-dried for storage as a lyophilized powder. Before use, PLGA nanoparticles were washed and dissolved at a final concentration of 5 mg/mL in PBS for injection.

### Interventions

PLGA-IMPs were diluted to 5 mg/ml concentration in 1X PBS and 200 µl per mouse (1 mg) per day was delivered intravenously via tail vein injection. The particles were administered daily for up to 4 days, with the first dose given 2–3 h following skin application of NM. For Treg inactivation, 500 µg of PC-61.5.3 anti-CD25 (BioXCell, Lebanon, NH) or control IgG isotype-matched antibody HRPN (BioXCell) were given via intraperitoneal injection 4 days and 2 days before NM application.

### Skin monitoring

Mice were observed daily after induction of NM skin injury while they were under inhalational anesthesia using a VetEquip V1 small animal anesthesia system with isoflurane. The following measurements were taken for up to 5 days: The bifold skin thickness within the 12 mm template area was measured using a digital caliper (Mitutoyo, PK0505CPX). Photographs of the injured area were taken using an iPhone camera mounted on a box. Mice were weighed using a mini scale. Wound areas were traced, analyzed, and quantified using ImageJ software (National Institutes of Health, Bethesda, MD).

### RNA expression and cell type enrichment

Skin injury and wound healing–related gene expression profiling was measured with real-time quantitative PCR. Skin was collected from euthanized mice under Avertin anesthesia. Total RNA was extracted from mouse skin tissue using TRIzol reagent (Invitrogen,15596026), per manufacturer’s instructions. TaqMan gene expression assays were used for measuring the relative expression levels of interleukin 1β (IL-1β; Mm00434228_m1), tumor necrosis factor-alpha (TNF-α; Mm99999068_m1), matrix metallopeptidase9 (MMP9; Mm00442991_m1), and nitric oxide2 (iNOS; Mm01309902_m1). 18 s (Hs99999901_s1) was used as the control housekeeping gene. 100 ng of each RNA was amplified using TaqMan Fast Virus 1-Step Master Mix (4444434). The relative expression was calculated using the ddCt method as fold change compared to controls.

Bulk RNA-seq counts (GEO accession number GSE218810) were created from skin biopsies taken as a part of our clinical trial NCT02968446^20^. Biopsies were collected before NM exposure and 72 h post-exposure were normalized and subjected to the “raw Enrichment Analysis” from the “xCell”^34^ package in R for cell type enrichment analysis of curated cell type gene sets. Enrichment scores were then submitted to the interactive xCell heatmap viewer https://comphealth.ucsf.edu/app/xcellview/. Weak signatures were filtered out and enrichment scores were percentiled. The column dendrogram was generated using Euclidean distance and Ward.D linkage.

### Flow cytometry

Mouse skin samples were mechanically disrupted followed by digestion using Liberase TL (Sigma, 1 h incubation at 37 °C) at concentration 0.25 mg/mL dissolved in RPMI-1640 with l-glutamine (Corning), supplemented with 1 mM sodium pyruvate, MEM nonessential amino acids, and 20 mM HEPES (Gibco). The resulting cellular suspension was filtered through 70 then 40 μM cell strainers and centrifuged at 500 × *g* for 10 min. at room temperature. The cell pellet was collected, washed with a PBS solution containing 5% FCS serum (PBS/FCS), and resuspended in fluorescence‐activated cell sorting (FACS) buffer (PBS with 2% fetal calf serum), and cells counted. Spleens were triturated, passed through 100 μm cell strainers, spun at 500 × *g* for 10 min, and washed with PBS/FCS. Red blood cells were lysed with Tris-ammonium chloride (0.16 M) for 5 min at room temperate, washed with PBS/FCS, and spun at 500 × *g* for 10 min. The cell pellet was resuspended in FACS buffer. The numbers of each subpopulation in the skin and spleen were determined by multiplying the percentage of lineage marker–positive cells by the total number of mononuclear cells isolated from the corresponding tissue.

Single cells were incubated with Fc block (anti-mouse CD16/32, 0.25 μg; eBioscience, San Diego, CA) for 30 min. at 4 °C. Cells were then washed with FACS buffer containing PBS with 2.5% (volume/volume) fetal bovine serum and 0.1% (weight/volume) NaN_3_ (Sigma‐Aldrich). Cells were then stained for surface markers (at a dilution range of 1:100 to 1:250) for 30 min at 4 °C using the specified antibodies. These antibodies included CD45, CD11b, CD11c, Ly6C, Ly6G, MHC class II, CD80, CD86, B220, CD3, CD4, CD8, CD49b, PD-1, CD122, IL-10, ST2, TGF-β, IL-6 and MARCO. Cells were then washed with PBS, and viability staining was performed using the LIVE/DEAD fixable dead cell stain kit (Invitrogen, Carlsbad, CA). Following viability staining, cells were washed with PBS and were either resuspended in FACS buffer for flow cytometric analysis or were subjected to intracellular staining to detect inducible nitric oxide synthase (iNOS), CD206, or FoxP3. For intracellular staining, cells were fixed and permeabilized using the FoxP3 staining buffer kit (eBioscience) and then intracellularly stained. Briefly, cells were incubated in fixation buffer from eBioscience for 45 min at 4 °C, washed with Permeabilization buffer, stained with a cocktail of antibodies specific for intracellular cytokines for 45 min at 4 °C, washed again and analyzed on a cytometer. As controls, fluorescence minus one was used to place the gates for analysis.

For flow cytometric analysis, cells were first gated according to forward and side scatter and then restricted to single cells and live cells. Tissue infiltrating myeloid cells were identified as CD45^+^CD11b^+^CD3^−^ and infiltrating lymphoid cells as CD45^+^CD11b^−^CD3^+^ for T cells and CD45^+^CD3^−^CD11b^−^B220^+^CD11c^−^ for B cells. On the infiltrating lymphoid population, cells were gated on CD3^+^CD4^+^CD8^−^ or CD3^+^CD8^+^CD4^−^ to evaluate the different T lymphocyte subpopulations. For infiltrating myeloid cells, Ly6G^+^ neutrophils were first gated and excluded from the infiltrating myeloid subpopulations. The Ly6G^−^ myeloid cells were divided into CD11c^+^ myeloid dendritic cells (mDCs) and CD11c^−^ monocytes/macrophages. Finally, the monocytes/macrophages were further divided into Ly6C^hi^ inflammatory monocytes and Ly6c^lo^ noninflammatory monocytes. From these subpopulations, expression of CD86 and MHC II were evaluated. Expression of iNOS, eGR2, and CD206 were evaluated on the monocyte/macrophage subpopulation.

Mouse-specific antibodies applied to the mixture of cell types segregated as described above are listed in the key resources Table 1 (Anti-mouse antibodies used for flow cytometry). A 6-laser Fortessa flow cytometer (BD Biosciences) was used to enumerate cell populations, and the data was analyzed using FlowJo software (TreeStar, Ashland, OR).

**Table 1:** Anti-mouse antibodies used for flow cytometry

SPECIFICITY	SOURCE	CLONE
CD45	BD Biosciences	30-F11
CD11b	BioLegend	M1/70
CD11c	BioLegend	N418
CD122	BioLegend	TM- β1
CD25	BioLegend	3C7
CD3	BioLegend	500A2
CD4	BioLegend	RM4-4
CD44	BioLegend	IM7
CD45	BioLegend	30-F11
CD49b	Biolegend	DX5
CD62L	BioLegend	MEL-14
CD69	BioLegend	H1.2F3
CD8	BioLegend	53-6.7
CD80	BioLegend	16-10A1
CD86	BioLegend	GL-1
FoxP3	BioLegend	FJK-16s
I-A/I-E	BioLegend	M5/114.15.2
IL-10	BioLegend	JES5-16E3
IL-6	BioLegend	MP5-20F3
Ly6C	BioLegend	HK1.4
Ly6G	BioLegend	1A8
MARCO	R&D Systems	FAB2956A
PD-1	BioLegend	29F.1A12
ST2	BD Biosciences	U29-93
TGF-β	BioLegend	TW7-16B4

### Histology and microscopy

Half of the 12 mm wound area from euthanized mice were fixed in 10% formalin solution for 24 h. Samples were then embedded in paraffin, sectioned at 4-μm thickness, and stained with hematoxylin and eosin Y. Slides were scanned in a brightfield microscope and images were captured using a mounted camera. For immunohistochemistry, slides were incubated with anti-human MARCO (Abcam, ab231046), anti-mouse MARCO (Abcam, ab256822) antibodies, or isotype-matched control IgG (eBioscience, San Diego, CA) followed by HRP-conjugated secondary antibody, which was visualized with diaminobenzidine substrate, and counterstained with hematoxylin.

### Statistics

Data are presented as means ± SEM. Two-tailed Student’s *t*-test or Mann Whitney *U* test was used for comparisons between 2 groups. If experiment involved more than three groups, 1-way ANOVA followed by Tukey or Sidak’s analysis was used to examine for statistical significance. The Chi Square analysis was used for categorical variables. A *p*-value less than 0.05 denotes a statistically significant difference. The Pearson correlation for continuous variables and the Spearman correlation for ordinal variables were used to evaluate relationships between two variables. Data were analyzed and graphs were created using GraphPad prism (GraphPad Prism Software version 7.03, GraphPad Software Inc.). *p < 0.05, **p < 0.01, ***p < 0.001, and ****p < 0.0001. The percent variation of skin thickness, weight, and wound area was calculated and graphed using GraphPad Prism software. The built-in statistics in Prism software was used to calculate the significance of the results. Fold change values of RNA expression were plotted using GraphPad Prism software, and statistical significance was calculated with built-in One-way ANOVA method.

### Study approval

All animal studies were conducted in accordance with NIH guidelines for the care and use of laboratory animals and protocols were approved by the Institutional Animal Care and Use Committee of Northwestern University. Studies involving human subjects were approved by the IRB of Northwestern University, all participants provided written informed consent, and study complied with all relevant ethical regulations including the Declaration of Helsinki.

## Supplementary information

Supplementary Information

## Data Availability

All data generated or analyzed during this study are included in this published article [and its supplementary information files]. Bulk RNA-seq data are deposited to Gene Expression Omnibus (GEO) accession number GSE218810.
