# Supplementary material for: Attenuation of skin injury by a MARCO targeting PLGA nanoparticle
Source: NPJ Regen Med. 2024 Dec 6;9:37. doi: 10.1038/s41536-024-00381-z (PMC11621362; doi:10.1038/s41536-024-00381-z)
Supplement: Supplementary file 1 — Supplementary Information [file 41536_2024_381_MOESM1_ESM.pdf]

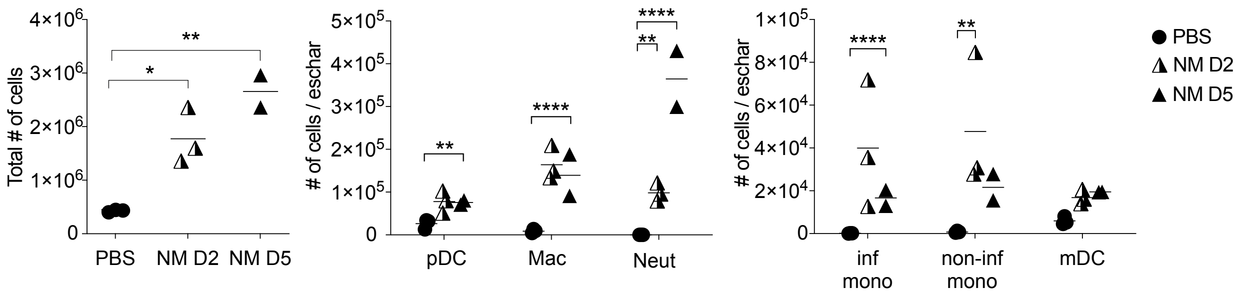

Supplementary Figure 1. Time course study showing the immune filtration on day 2 and day 5 collected skin. The graphs show the significant infiltrate increase on skin, collected 2 days post NM insult. *$p<0.05$, **$p<0.01$, ***$p<0.001$, ****$p<0.0001$

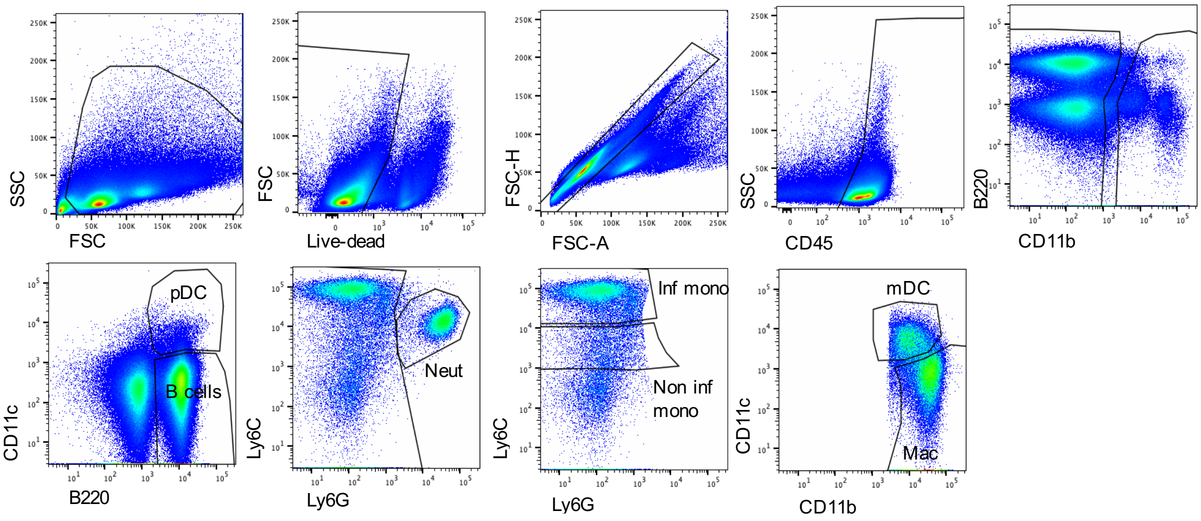

Supplementary Figure 2. Gating strategy for APC subsets. A NM+PLG mouse spleen sample is shown as a representative to demonstrate the sequential gating strategy used to discriminate neutrophils (Neut) (LD$^-$/CD45$^+$/CD11b$^+$/B220$^-$/Ly6G$^+$/Ly6C^{int}), B cells (LD$^-$/CD45$^+$/CD11b$^-$/B220$^+$/CF11c$^-$), plasmacytoid DC (pDC)(LD$^-$/CD45$^+$/CD11b$^-$/B220$^+$/CD11c$^+$), myeloid DC (mDC)(LD$^-$/CD45$^+$/CD11b^{int}/B220$^-$/Ly6G$^-$/Ly6C$^{low\text{-}int}$/CD11c$^+$), inflammatory monocytes (LD$^-$/CD45$^+$/CD11b$^+$/B220$^-$/CD11c$^-$/Ly6G$^-$/Ly6C^{hi}), and non-inflammatory monocytes (LD$^-$

$/CD45^{+}/CD11b^{+}/B220^{-}/CD11c^{-}/Ly6G^{-}/Ly6C^{int}$). FSC, forward scatter; SSC, side scatter; FSC-H, forward scatter height; FSC-A, forward scatter area; DC; dendritic cells.

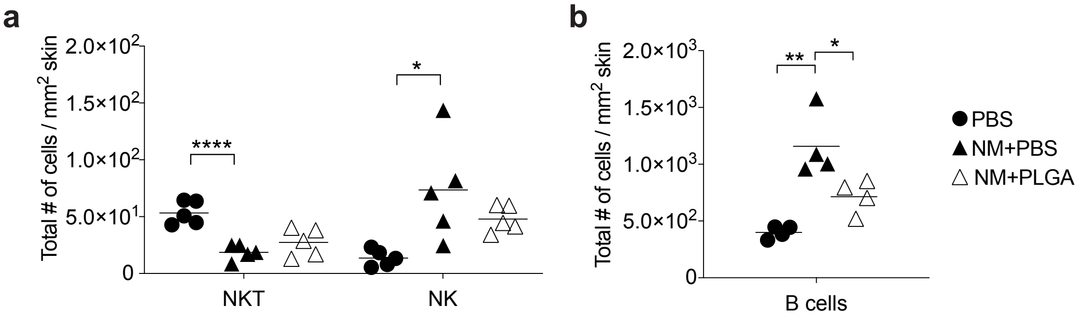

Supplementary Figure 3. Significant modulation of natural killer cells **(a)** and B lymphocytes **(b)** in NM-induced skin lesions following PLGA nanoparticle treatment. *p<0.05, **p<0.01, ***p<0.001, ****p<0.0001

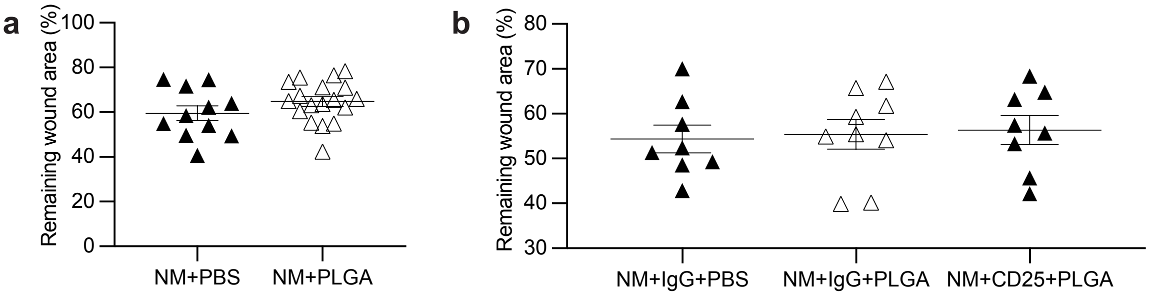

Supplementary Figure 4. Remaining wound area measurements on day 3 post NM injury. For proper comparison purposes we included in Panel **a**, the measurements from Day3 wounds of mice from Fig. 1c. Panel **b** shows the measurements of remaining day3 wounds from CD25 depletion experiments. No statistical significance is observed.
